# Macrolide Antibiotic Mediated Cardiac Arrhythmias: Emerging Concepts and Clinical Implications

**DOI:** 10.3390/biomedicines13061478

**Published:** 2025-06-16

**Authors:** Fatima Iqbal, Alyssa Derouen, Robin Ren, Adam M. Kaye, Shahab Ahmadzadeh, Sahar Shekoohi, Alan D. Kaye

**Affiliations:** 1 Department of Internal Medicine, University of Texas Health Science Center at Houston, Houston, TX 77030, USA; 2 Department of Anesthesiology, Louisiana State University Health Sciences Center Shreveport, Shreveport, LA 71103, USA; 3 Department of Pediatrics, University of Arkansas for Medical Sciences, Little Rock, AR 72205, USA; rren@uams.edu; 4 Department of Pharmacy Practice, Thomas J. Long School of Pharmacy, University of the Pacific, 751 Brookside Road, Stockton, CA 95207, USA; 5 Departments of Anesthesiology and Pharmacology, Toxicology, and Neurosciences, Louisiana State University Health Sciences Center Shreveport, Shreveport, LA 71103, USA

**Keywords:** macrolide, azithromycin, erythromycin, clarithromycin, arrhythmia, torsades

## Abstract

The macrolide class of antibiotics are widely utilized in clinical settings for a broad range of bacterial infections and have additional roles as immunomodulatory agents. Although efficacious with a good safety profile overall, they have been associated with prolongation of the QT interval and development of the polymorphic ventricular tachycardia, Torsades de pointes (TdP). In a 2020 scientific statement, the American Heart Association (AHA) classified azithromycin, clarithromycin and erythromycin as QT-prolonging drugs known to cause TdP and the online database, CredibleMeds, that maintains a list of drugs known to cause QT prolongation classifies these drugs as having an increased risk of QT prolongation. The mechanism of this risk has been delineated to involve macrolide binding to and a blockade of delayed rectifier potassium channels that conduct rapid potassium current, I_kr_, during repolarization, leading to prolonged repolarization and subsequent QT prolongation. Studies investigating this association have revealed variable results, with several suggesting that the risk of QT prolongation and TdP with macrolide use may be highly dependent on underlying patient risk factors and comorbidities. In the present investigation, we summarize current evidence on association of macrolide antibiotics, azithromycin, clarithromycin and erythromycin, with the development of QT prolongation and TdP, pathophysiology of and risk factors predisposing to development of these events, the role of implementation of strategies to reduce this risk and highlight emerging research.

## 1. Introduction

Since the advent of erythromycin over 50 years ago, macrolide antibiotics have become commonly utilized for a broad range of clinical conditions. This antibiotic class, which includes the FDA-approved agents’ erythromycin, azithromycin and clarithromycin, is active against Gram-positive bacteria, as well as several Gram-negative and atypical bacteria. As such, it has been utilized in the treatment of infections of the respiratory tract, soft tissue and genitourinary tract. In addition, research in recent years has highlighted the potent immunomodulatory effects of these antibiotics, and they play an important role in the treatment of chronic inflammatory diseases [1,2].

Although, overall, macrolides are classified as drugs with limited and tolerable side effects, they have been linked to QT prolongation and the development of polymorphic ventricular tachycardia, Torsades de Pointes (TdP). In May 2012, the FDA issued a warning regarding the risk of QT prolongation with Azithromycin use, based off of an observational study published the same year that found a small increase in risk of cardiovascular death in patients taking Azithromycin [3]. In March 2013, this warning was further strengthened by the FDA, with emphasis on the risk of fatal arrhythmias with azithromycin use, and the risk factors associated with this development were highlighted. Additionally, several case reports and observational studies have reported on the association between macrolides and the risk of QT prolongation and TdP [4,5,6,7,8,9,10,11]. Yet, results from other studies have found no association [12,13,14,15,16,17].

However, a scientific statement from the American Heart Association in 2020 included the macrolides azithromycin, erythromycin, and clarithromycin, as well as Roxithromycin, in the list of QT-interval-prolonging drugs known to cause TdP [18]. A 2013 review in the European society of cardiology classified erythromycin as a high-risk drug with frequent reports of TdP, while azithromycin and clarithromycin were classified as a moderate risk of TdP [19]. The Arizona center for education and research on therapeutics (AZCERT), which maintains a database, CredibleMeds, to keep track of QT-prolonging drugs, classifies azithromycin, clarithromycin and erythromycin as drugs with a known risk of TdP.

Yet, aside from these conflicting results, increasing bacterial resistance to antibiotics remains a looming concern, and the macrolide antibiotics represent indispensable weapons in an ever-dwindling arms race against bacterial pathogens. As such, the macrolides remain crucial drugs which cannot be relegated to the sidelines. Their use must then be deployed following careful consideration of the risk of the development of arrhythmias and adoption of careful monitoring when used to prevent adverse cardiac events.

In the present investigation, we describe the clinical applications of macrolides and summarize current literature on the association between macrolides and arrhythmias.

## 2. Use of Macrolides in the Clinical Setting and Mechanism of Action

The macrolide antibiotics include agents approved by the United States FDA, such as erythromycin, azithromycin, clarithromycin and fidaxomicin, as well as non-FDA-approved agents such as spiramycin and roxithromycin. The center for disease control and prevention (CDC) has identified that 34.9 million prescriptions for macrolides were dispensed in outpatient pharmacies in 2023, highlighting the extent of their clinical use.

Macrolides are active against most Gram-positive bacteria, several Gram-negative bacteria and certain atypical bacteria, including chlamydia. Compared to erythromycin, an older agent, both clarithromycin and azithromycin exhibit extended Gram-negative activity [20]. As such, these antibiotics are used in the treatment of a broad range of infections, from those of the respiratory tract and genitourinary tract to skin and soft tissue infections. FDA-approved indications for azithromycin use include the treatment of community-acquired pneumonia (CAP) in children and adults, upper respiratory infections such as acute otitis media and pharyngitis, sexually transmitted infections caused by chlamydia trachomatis and Hemophilus Ducreyi, and both treatment and prophylaxis for mycobacterium avium complex (MAC) in patients with HIV [21]. In addition, clarithromycin is a component of combination therapy for the treatment of Helicobacter-pylori-associated gastritis [22]. Macrolides also play a role in the management of certain skin conditions [23]. In particular, azithromycin is effective for the treatment of acne, while clarithromycin has shown efficacy in the treatment of leprosy caused by atypical bacteria (mycobacterium leprae) as a second-line agent when there is resistance to rifampin [23]. Fidaxomicin, a comparatively lesser-known macrolide antibiotic, is utilized in the treatment of clostridium difficile diarrhea [24].

In addition to their antibacterial properties, macrolides also possess immunomodulatory effects which have been investigated in the context of chronic inflammatory airway disease [25]. Prior observational studies and randomized clinical trials have demonstrated that macrolides reduce the number of exacerbations and improve quality of life in patients with diseases such as chronic obstructive pulmonary disease (COPD), cystic fibrosis and bronchiectasis [11,26,27,28,29,30]. These anti-inflammatory effects include the inhibition of proinflammatory cytokine release, decreased leukocyte adhesion, increased phagocytosis by macrophages, increased production of reactive oxygen species that are microbicidal as well as decreased release of inflammatory enzymes [25]. These anti-inflammatory effects stem from interference with cell signaling through the modulation of intracellular signaling pathways. These pathways involve the mitogen-activated protein kinase (MAPK) system, as well as the expression of transcription factors such as NF-kappa B which then affect processes such as inflammatory cytokine production and cell proliferation [31].

Conversely, the antibacterial effects of macrolides stem from their ability to inhibit bacterial protein synthesis, and they have thus been labelled as bacteriostatic agents. Their mechanism of action involves binding to the bacterial 50S ribosomal subunit at the peptide exit tunnel site, which subsequently prevents the translation of bacterial mRNA [22]. However, recent research has suggested that rather than being global inhibitors of protein synthesis, macrolides exert their effects based on specific nascent protein sequences, and have therefore been termed as modulators of protein translation [32].

## 3. Association Between Macrolide Use and Types of Arrhythmias

Several studies have previously evaluated the link between macrolides and arrhythmia and are summarized in Table 1. The results from these studies reveal conflicting outcomes.

Several studies have found an association between macrolides and the development of QT prolongation and arrhythmias. Mishra et al. demonstrated in their retrospective study that erythromycin use was associated with QT prolongation [6]. A pharmacovigilance analysis of the FDA adverse events reporting system (FAERS) also found a disproportionality signal for both TdP/QT abnormalities and ventricular arrhythmias/sudden cardiac death with both azithromycin and clarithromycin, while a disproportionality signal was only found for erythromycin with TdP and QT abnormalities [7]. Still, other cohort studies demonstrated that both azithromycin and clarithromycin may be associated with an increased short-term risk of arrhythmia following recent use of macrolides [8,33]. Similarly, a meta-analysis in 2015 further supported these findings as it found macrolide use to be associated with increased risk of ventricular arrhythmias and sudden cardiac death [9].

However, contrary to these findings, several studies have also reported a lack of association between macrolide use and arrhythmia risk [12,13]. Significantly, a few studies demonstrated that when controlled for underlying comorbidities and patient demographics, the association of macrolides with arrhythmias was no longer present [14,16]. This finding would explain that underlying comorbidities may explain the association between macrolides and arrhythmias. For example, patients with pneumonia may be predisposed to arrhythmia, and since macrolides are used to treat pneumonia, an association may be present when this is largely due to the underlying comorbidity rather than the macrolide itself. However, a cohort study by Rao et al. that compared the risk of arrhythmias with azithromycin to amoxicillin found a statistically significant association of azithromycin with arrhythmias even when controlling for covariates [8].

While these studies provide insights into the association between macrolides and arrhythmias, it is important to also note that the true incidence of events such as TdP and sudden cardiac death is difficult to accurately quantify. TdP, when occurring in an unmonitored setting, may often go unnoticed, or, if it culminates in sudden cardiac death, the death may not always be reported or an autopsy, which may rule out other cardiac causes, may not be performed. A Danish study by Risgaard et al., investigating the association between sudden cardiac death and pharmacotherapy, found antibiotics to be amongst the most frequently associated drugs with sudden cardiac death, while also noting that this association may be stronger if not limited by underreporting, as previously mentioned [34]. This is further supported by the findings of Molokhia et al., who found the rate of reporting for drug-induced LQTS to be low, at 7.5% [35].

## 4. Mechanisms Underlying Development of QT Prolongation and Torsade de Pointes by Macrolides

The QT interval, as noted on the electrocardiogram, represents ventricular depolarization and repolarization. Prolongation of the QT represents abnormalities in the depolarization and repolarization phase of the cardiac cells, through interference with the ion channels that allows for these events to occur [36]. QT prolongation is a well-established risk factor for the development of TdP as well as sudden cardiac death (SCD) [37]. QT prolongation that results from the use of medications has been termed acquired long QT syndrome (aLQTS), as opposed to QT prolongation resulting from genetic mutations, aptly termed congenital long QT syndrome (cLQTS). Macrolides have been associated with the development of aLQTS, and subsequently with torsades de pointes.

The repolarization phase of the cardiac action potential is characterized by the inward movement of sodium and calcium ions and the outward movement of potassium ions [36]. The voltage-gated channels that facilitate potassium movement are the delayed rectifier potassium channels. It is the current through these channels (I_k_) that is the predominant current responsible for repolarization [9]. These delayed rectifier potassium channels create a current that has both a slow component (I_KS_) and a rapid component (I_KR_). I_kr_ is conducted through channels made up of proteins, such as Kv11.1, encoded by KCNH2, alternatively known as the human ether-go-go gene (HERG) [38]. Notably, mutations in genes such as KCNH2 and others that encode subunits forming these channels are implicated in congenital long QT syndromes [36]. Drug-induced QT prolongation then occurs when I_kr_ channels are inhibited by binding of drugs to the Kv11.1 protein, resulting in prolonged repolarization [39], which subsequently permits the occurrence of events termed early afterdepolarizations (EADs) [36]. An EAD represents an abnormal depolarization that subsequently results in interruption or reversal of the repolarization phase [40], as well as alteration of the cardiac membrane threshold potential. This alteration allows for the development of a spontaneous action potential, termed a triggered response, which can then give rise to premature ventricular complexes (PVCs) and subsequently allow for the development of tachyarrhythmias [41]. This same mechanism has been highlighted in the development of QT prolongation by macrolides, wherein inhibition of the I_kr_ channels leads to QT prolongation [42]. Notably, Yang et al. found that erythromycin also led to increased late sodium currents (I_Na-L_) through the phosphoinositide 3-kinase pathway, with a similar result of QT prolongation [43].

## 5. Risk Factors for Development of Arrhythmias with Macrolide Use

**Figure 1:**
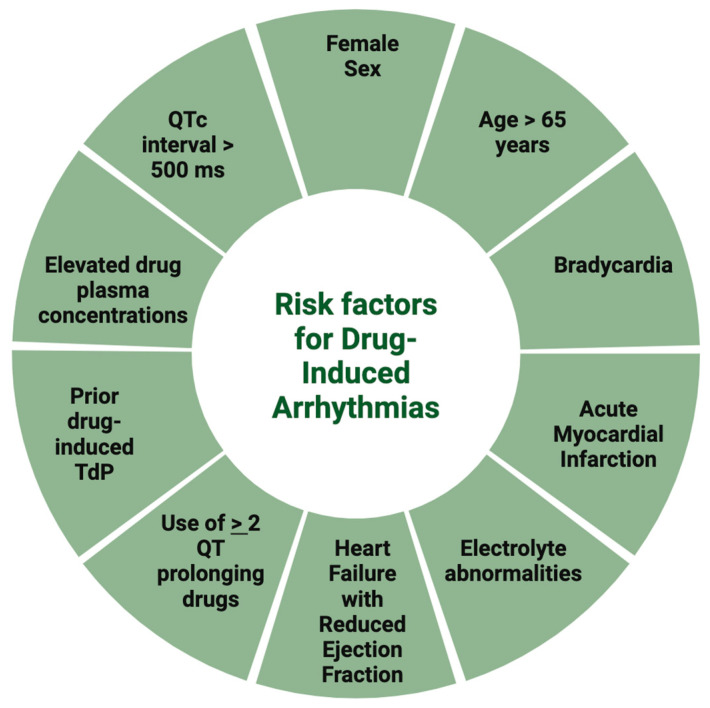
Risk factors associated with the development of drug-induced arrhythmia.

Several risk factors have previously been implicated in the development of drug-induced QT prolongation, Figure 1. In fact, prior research has emphasized that the presence of such risk factors is almost prerequisite for the development of drug-induced arrhythmias, and when controlled for many of the underlying patient comorbidities and demographics, the risk of arrhythmias with macrolide use was not statistically significant [16]. Table 2 summarizes the strategies used for confounding in studies evaluating risk of arrhythmias with macrolide use.

A case series of TdP cases, in which half of cases were associated with macrolide use, found older age, female sex, concomitant drug use and comorbid disease to be risk factors for the development of macrolide-associated TdP [44]. These results were congruent with another analysis of reports of TdP associated with use of non-cardiac drugs that found female sex to be the most common risk factor, with all patients possessing at least one risk factor and 71% of patients possessing two or more risk factors [45]. Similarly, a prospective study of patients admitted to critical care units examined the association between AHA indications for QT interval monitoring, which are based on risk factors, and the QT interval found that the odds of QT interval prolongation were higher in patients with an increased number of indications: 9-, 7- and 3-fold higher in patients with three, two and one indications, respectively, as compared to patients with no indications [46].

The mechanisms that underlie the enhanced risk due to these risk factors have previously been elucidated. Zeitler et al. proposed that women likely have an increased susceptibility to TdP with antiarrhythmic drug use due to hormonal effects, such as I_kr_ blockade by estrogen, and differential ion channel expression [47]. This concept can be extrapolated to macrolide use, which similarly results in a blockade of I_kr_.

With hypokalemia, multiple mechanisms have been highlighted, including inactivation of Kv11.1 channels, increased blockade of channels by drugs in low potassium concentration and potential activation of CaM kinase with increases in late sodium currents [36]. Notably, ion channel remodeling in heart failure, which is ultimately maladaptive, results in the downregulation of potassium channels and decreased I_kr_, resulting in prolonged action potentials and the increased arrhythmogenic risk that has been observed in heart failure [48].

Genetic variants of genes implicated in cLQTS, such as KCNE1, KCNE2, KCNH2, KCNQ1 and SCN5A, have also been suggested to modulate the risk for the development of drug-induced TdP [49,50,51,52,53]. These studies found genetic mutations in cases of aLQTS ranging from rates of 5 to 12% [53]. A recent study similarly discovered that genetic variants of the cardiac sodium channel gene (SCN5A) are likely implicated in one’s predisposition to drug-induced arrhythmias [54]. These variants, which lead to increased I_Na-L_, demonstrated normal repolarization at baseline, but were found to have increased levels of I_kr_, a likely compensatory response to maintain adequate repolarization. However, I_kr_ blockade with Dofetilide resulted in increased action potential duration (APD) and afterdepolarization in cells with variants, compared to controls, highlighting that when challenged with proarrhythmic drugs, these genetic variants likely result in increased sensitivity to proarrhythmic effects of I_kr_-blocking drugs [54]. Additionally, variations in the cytochrome P450 system have also been previously investigated regarding their association with drug-induced QT interval prolongation. Variants of CYP2C19, CYP3A4 and CYP2C9 have all previously been found to increase the risk of QT prolongation or the development of torsades de pointes in the context of antibiotic use (neimeijer).

## 6. Minimizing the Risk of Cardiac Arrhythmias

To minimize the risk of macrolide-associated arrhythmias, attention to risk factors and interventions aimed at minimizing this risk are crucial, as discussed below.

### 6.1. History

Obtaining a thorough history is of utmost importance, with particular attention paid to comorbidities and factors that can augment the risk of the patient developing arrhythmias, such as concomitant heart failure, the use of diuretics, bradycardia, the presence of hepatic or renal disease, family history of long QT syndrome, or simultaneous use of other medications that have been known to prolong the QT interval [27,55].

### 6.2. Electrocardiogram Monitoring

ECG and QT measurements have variability over time and can be affected minute to minute by a multitude of dynamic factors, including alterations in the autonomic nervous system such as stress, breathing, and posture. Electrolyte shifts in potassium, magnesium or calcium levels can alter repolarization. Ischemia or hypoxia can result in a reduction in myocardial oxygen supply affecting the QT interval. Fluid status, heart rate fluctuations, and electrode placement artifact can alter QT intervals. Therefore, medications cannot be looked at in isolation vis-à-vis clinical context and real-time tracking of ECG. It should be noted that Al-Khatib et al. previously recommended that an ECG should be offered to all patients before and after starting a QT-prolonging drug, while Shah et al. suggested that it should only be offered to patients with QT prolongation risk factors [55,56]. The ECG obtained should be of good quality, with a stable baseline and without significant artifact. The QT interval should be calculated and an average value obtained from 3 to 5 cardiac cycles. The rest of the ECG should be evaluated and assessed for presence of myocardial infarction, bradycardia, cardiomyopathy, or other conditions. Given natural variation in QT interval at different heart rates, Bazett’s formula should be applied to calculate QTc. A QTc value > 500 ms is linked to a significantly increased risk for TdP, with the risk of TdP increased by approximately 5–7% for every 10 ms prolongation of the QTc interval [57].

Shah advises that the QTc interval via ECG should be monitored at baseline, at steady state after treatment initiation (usually after around 4–5 half-lives), and at each increase in dosage, when there is an intercurrent change in risk, and if the patient experiences common symptoms of arrhythmias, such as dizziness, palpitations, or syncope. If the patient has a prolonged QTc on baseline ECG, then treatment should be approached carefully. If the patient develops a QTc interval of 500 ms or has a significant increase (>50 ms), electrolyte levels should be checked and corrected. If QTc remains prolonged, a decrease in dosage or drug discontinuation should be considered [55,57].

### 6.3. Clinical Decision Support System

Tisdale et al. previously examined whether a clinical decision support system (CDSS) incorporated into the electronic medical record could be beneficial [58]. This method, which utilized a validated risk score to assess patient risk, was followed by a computer alert to notify the pharmacist to then contact the provider to discuss alternative treatments and the potential incorporation of more intense monitoring. This intervention was found to decrease the risk of QT interval prolongation in hospitalized patients with risk factors for torsades de pointes. To prevent alert fatigue, incorporation of a risk assessment score and alert pop-ups only for patients with a moderate to high-risk QTc were instituted [58]. However, while helpful in directing attention to the side effects associated with macrolide use, the incorporation of CDSS has several limitations, including a lack of applicability in resource poor settings, as well as lack of standardization as well as incorporation across electronic medical records.

### 6.4. Pharmacogenetic Testing

In recent years, there has also been increasing emphasis on the role of pharmacogenetic testing to evaluate the risk of drug-induced LQTS [59]. A secondary analysis by Strauss et al. of a randomized, placebo-controlled trial found that a composite genetic QT score, based on 61 common genetic variants, was significantly associated with drug-induced QT prolongation [60]. However, further research is currently needed to support the implementation of this intervention.

## 7. Evaluation of Risk

**Figure 2:**
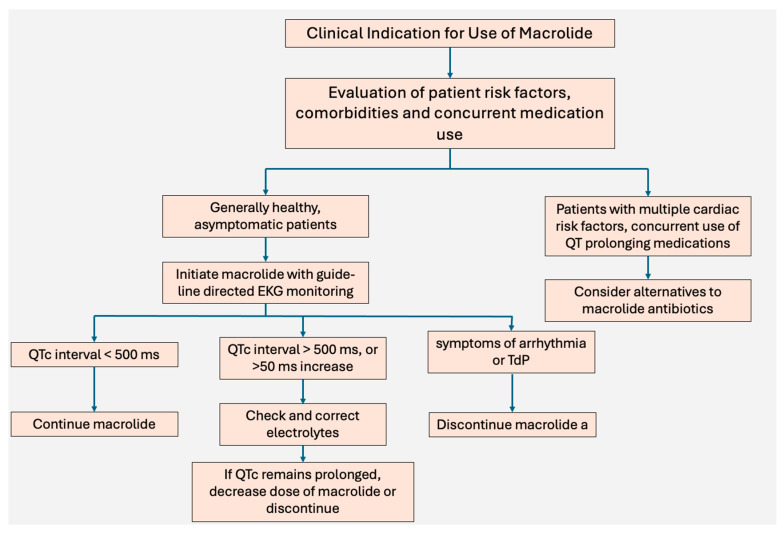
Proposed algorithm for evaluation of the risk–benefit profile of macrolide initiation.

Figure 2 shows a proposed algorithm for comprehensive evaluation of the arrhythmic risks associated with macrolide use, that can assist with clinical decision making on whether it is safe to initiate or continue use of macrolide antibiotics. We recommend a thorough baseline evaluation of patient risk factors, with particular attention to ones discussed previously in Figure 1. If patients possess multiple risk factors, then alternative therapy should be sought to avoid the development of QT prolongation. Additionally, if at any point in therapy, the QTc interval is found to be greater than 500 ms, there is evidence of TdP or the patients experiences symptoms of arrhythmia, i.e., syncope, lightheadedness or dizziness, the antibiotic should be discontinued.

## 8. Conclusions

The macrolide class of antibiotics constitute a cornerstone of treatment of numerous bacterial infections and have additional roles as anti-inflammatory agents in the management of chronic inflammatory diseases.

However, their association with cardiac rhythm abnormalities, namely QT interval prolongation with subsequent predisposition to the development of the polymorphic ventricular tachycardia, Torsade de Pointes (TdP), has previously raised concerns regarding their clinical safety.

The current literature shows variable results, with some studies finding a significant association between the use of macrolides and the development of QT prolongation and TdP. However, factors that have been highlighted that may influence these results include the potential of confounding by underlying comorbidities as well as the underreporting of events such as torsades de pointes and sudden cardiac death in unmonitored settings. In addition to well known risk factors such as female sex, older age, heart failure and electrolyte abnormalities, genetic variants of key genes involving ion channel proteins may also play a role in modulating the risk of prolonged QT and arrhythmias associated with drugs such as macrolides, although further research investigating this association is still ongoing.

Medications all have known risks, possible risks, and conditional risks. In this regard, the totality of potential consequences related to macrolide antibiotic therapy cannot be automatically identified as the cause of further deterioration in acute illness. Thus, patients often require macrolides, which are among the most efficacious antibiotics and should not be denied as a result of recent use of reasonable doses or other QT-prolonging medications. However, highly torsadogenic medications such as methadone, citalopram, and others should be used with caution in patients, while many others can be used confidently in many stable patients. 

Analysis of the current literature suggests that there is a need for greater attention to risk factors predisposing a patient to the development of QT prolongation with macrolide use, as well as the implementation of steps to reduce this adverse effect, mainly through appropriate electrocardiogram monitoring as well as the consideration of tools such as clinical decision support systems. The use of macrolides should occur following a careful assessment of patient risk factors and underlying comorbidities to evaluate the risks and benefits of macrolide use. Future research regarding the role of genetic variants and their association with drug-induced long QT syndrome and arrhythmias would undoubtedly provide greater insights and offer another possible intervention to mitigate this risk.

## Figures and Tables

**Table 1:** Association of macrolides with QT prolongation and arrhythmias.

Author	Groups Studied and Intervention	Results and Findings	Conclusions
Mishra et al., 1999 [6]	A single-institution prospective analysis of patients undergoing treatment for community-acquired pneumonia comparing ECG measurements in groups treated with 500 mg IV erythromycin vs. 500 mg IV erythromycin and 750 mg IV cefuroxime.	IV erythromycin caused increased heart rate and QTc interval prolongation that was most prominent at 15 min into the infusion.	One standard dose of erythromycin is enough to produce QTc interval prolongation.
Raschi et al., 2013 [7]	A pharmacovigilance analysis of the FDA Adverse Drug Reporting Database (FAERS) was carried out from 2004 to 2011 to evaluate the association of macrolides with arrhythmias.	The reporting odds ratio (ROR), 95% CI, for azithromycin and TdP + QT abnormalities was 5.69 (4.43–7.31), for clarithromycin 6.23 (5.01–7.74) and for erythromycin 5.28 (3.35–8.32).	Azithromycin, clarithromycin and erythromycin are associated with increased risk of QT abnormalities and TdP.
Rao et al., 2014 [8]	A cohort study of United States Veterans that compared the risk of arrhythmia with Azithromycin use compared to amoxicillin use.	Azithromycin was associated with increased risk of serious arrhythmia when compared to amoxicillin during first five days of treatment, [hazard ratio = 1.77, 95% CI, 1.20–2.62].	Azithromycin was associated with a statistically significant increased risk of serious arrhythmias for the first five days of treatment when compared to amoxicillin.
Cheng et al., 2015 [9]	A meta-analysis of thirty-three studies, involving population-based cohorts, case–control studies and randomized controlled trials was performed to evaluate the relative risk for sudden cardiac death (SCD) and VTA	When compared to no macrolide use, macrolide use resulted in increased risk of SCD or VTA (RR: 2.42; 95% CI: 1.60 to 3.63; *p* < 0.001).	Use of macrolides resulted in increased risk of SCD or VTA.
Chou et al., 2015 [10]	A Taiwanese nationwide, cohort study involving 10 648 100 patients evaluated the risk of ventricular arrhythmia with use of azithromycin and clarithromycin compared to amoxicillin-clavulanate, following 7 days of antibiotic initiation.	Compared to amoxicillin-clavulanate, azithromycin was associated with an increased risk of ventricular arrhythmia, adjusted ORs 4.32 (95% CI, 2.95–6.33). No association was noted with clarithromycin.	Compared to amoxicillin-clavulanate, azithromycin was associated with a significantly increased risk of ventricular arrhythmias.
Wong et al., 2016 [33]	A population-based study that compared short-term and long-term cardiovascular outcomes (myocardial infarction, arrhythmia, stroke) in clarithromycin and amoxicillin use	Compared to amoxicillin, clarithromycin was associated with increased short-term (14 days) risk of arrhythmia, 2.22 95% CI (1.22 to 4.06).	Clarithromycin carries a higher risk of short-term but not long-term risk of arrhythmia compared to amoxicillin.
Trac et al., 2016 [12]	A Canadian cohort study that compared the risk of ventricular arrhythmia within 30 days of prescription of macrolide antibiotics compared to non-macrolide antibiotics in adults over >65 years.	Macrolide antibiotics were not associated with an increased risk of ventricular arrythmia compared to non-macrolide antibiotics (0.3% in both groups with relative risk (RR) of 1.06, 95% CI 0.83–1.36).	Warnings from the US Food and Drug Administration (FDA) regarding macrolide use and QT interval prolongation and fatal ventricular arrhythmias may be overstated.
Berni et al., 2017 [13]	A UK-based cohort study that compared the risk of arrhythmias with use of clarithromycin as compared to other antibiotics.	clarithromycin did not result in an increased risk of arrhythmia compared to all antibiotics, for upper or lower respiratory tract infections, [adjusted hazards ratio, aHR 0.88, 95% CI 0.76–1.01) and, [aHR 1.39, 95% CI 0.80–2.41].	Clarithromycin did not result in an increased risk of arrhythmias when used for either upper or lower respiratory tract infections.
Trifiro et al., 2017 [14]	A nested case–control study evaluating the risk of ventricular arrhythmia associated with azithromycin used compared to amoxicillin or nonuse of antibiotic from 1997 to 2010.	Compared to nonuse of antibiotics, azithromycin use was associated with increased risk of ventricular arrhythmia, [adjusted odds ratio, aOR, 1.97, 95% CI 1.35–2.86]. Compared to amoxicillin use, there was no significant increased risk, [aOR 0.90, 95% CI 0.48–1.71].	Confounding due to underlying comorbidities may explain the association of azithromycin with ventricular arrhythmias.
Gorelik et al., 2018 [15]	A meta-analysis of thirty-three studies, involving 22,601,032 subjects was carried out. The odds ratio for arrhythmia, cardiovascular death and myocardial infarction (MI) was calculated.	Using a random-effects model, macrolide use was not associated with an increased risk for short-term arrhythmia (OR, 1.20 [95% CI, 0.91 to 1.57]).	Macrolide antibiotics as a group were not associated with a significant risk for arrhythmia or cardiovascular mortality.
Polgreen et al., 2018 [16]	A retrospective analysis of Medicare beneficiaries that compared outcomes such as ventricular arrhythmia, acute myocardial infarction and death between azithromycin, clarithromycin, levofloxacin, moxifloxacin, doxycycline and amoxicillin-clavulanate.	The unadjusted odds ratio (OR), 95% CI, for ventricular arrhythmias were 1.41 (1.14–1.73) and 1.63 (0.85–3.13) for azithromycin and erythromycin, respectively. The adjusted odds ratio, 95% CI, for ventricular arrhythmias were 1.13 (0.92–1.42) and 1.19 (0.62–2.28) for azithromycin and erythromycin, respectively.	Adjusting for comorbidities and patient demographics eliminates the statistically significant association of azithromycin and erythromycin with ventricular arrhythmias.
Postma et al., 2019 [17]	A multicenter trial involving seven teaching hospitals in the Netherlands from February 2011 to October 2013, comparing three empiric antibiotic courses (beta-lactam monotherapy, beta-lactam/macrolide therapy, and fluoroquinolone monotherapy) in the treatment of CAP.	In the sub-distribution hazard ratios, azithromycin, clarithromycin and erythromycin were not significantly associated with new or worsening arrhythmia.	No association was found between erythromycin, azithromycin and clarithromycin and arrhythmias.

**Table 2:** Confounding control strategies across studies.

Rao et al., 2014 [8]	Inverse probability weights (IPTW) for assignment into 3 exposure groups using covariates, including (race, age, sex), indication for antibiotics, comorbidities including cardiac morbidities, laboratory findings, and medication.
Chou et al., 2015 [10]	Multiple propensity scores, then included in a multivariable logistical regression analysis, were calculated by considering covariates such as indications of antibiotic use, comorbidities, concomitant medication, and health resource utilization
Wong et al., 2016 [33]	Propensity scores were calculated based on covariates and then used for subgroup analysis.
Polgreen et al., 2018 [16]	Adjusted regression logistic regression models were generated using patient comorbidities, medications, procedures, demographics, insurance status, number of visits and the influenza rate.
Postma et al., 2019 [17]	Adjusted hazards ratio were calculated based on confounders such as number of cardiac comorbidities and smoking history.
